# The Cup Anemometer, a Fundamental Meteorological Instrument for the Wind Energy Industry. Research at the IDR/UPM Institute

**DOI:** 10.3390/s141121418

**Published:** 2014-11-12

**Authors:** Santiago Pindado, Javier Cubas, Félix Sorribes-Palmer

**Affiliations:** 1 Instituto Universitario de Microgravedad “Ignacio Da Riva” (IDR/UPM), Universidad Politécnica de Madrid, ETSI Aeronáutica y del Espacio, Pza. Del Cardenal Cisneros 3, Madrid 28040, Spain; E-Mails: j.cubas@upm.es (J.C.); felix.sorribes@upm.es (F.S.-P.); 2 Departamento de Infraestructura, Sistemas Aeroespaciales y Aeropuertos, Universidad Politécnica de Madrid, ETSI Aeronáutica y del Espacio, Pza. Del Cardenal Cisneros 3, Madrid 28040, Spain

**Keywords:** cup anemometer, AEP, rotor dynamics, calibration, anemometer performance, anomaly detection

## Abstract

The results of several research campaigns investigating cup anemometer performance carried out since 2008 at the IDR/UPM Institute are included in the present paper. Several analysis of large series of calibrations were done by studying the effect of the rotor's geometry, climatic conditions during calibration, and anemometers' ageing. More specific testing campaigns were done regarding the cup anemometer rotor aerodynamics, and the anemometer signals. The effect of the rotor's geometry on the cup anemometer transfer function has been investigated experimentally and analytically. The analysis of the anemometer's output signal as a way of monitoring the anemometer status is revealed as a promising procedure for detecting anomalies.

## 1. Introduction

At present, the use of wind speed anemometers (cup, propeller or sonic anemometers) is very common, their applications having spread from sectors such as meteorology or wind energy to others where the effect of the wind should be taken into account (moving bridges in civil engineering, big cranes, *etc.*). Nevertheless, the wind energy industry can still be considered as the biggest consumer of anemometers worldwide.

**Figure 1.**
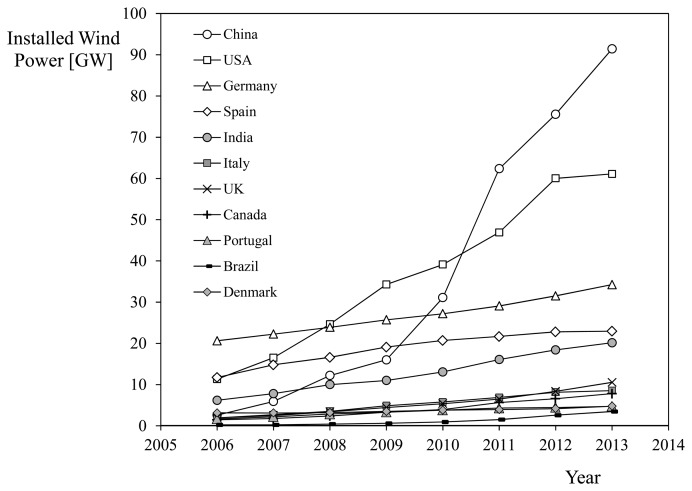
Installed wind power per country from 2006 to 2013. The graph includes data from some of the biggest producers in the world (sources: Global Wind Energy Council; US Energy Information Administration).

Leaving aside the importance of having the most accurate instruments (as the wind power is proportional to the third power of the wind speed [1]), the wind energy sector is extremely concerned with two aspects that, despite technological advances such as LIDAR and SODAR [2–5], require the use of anemometers: wind energy production forecasting in the field, and wind turbine performance control [6]. Over recent decades the wind energy sector has been openly supported by governments (Germany, Denmark, Spain…), concerned about clean energies and reducing their fossil fuels dependence [7]. In addition, new strong players in this industry like China, U.S.A., Brazil or India are now being very active, with large figures in terms of installed wind power and growing adoption rates (see Figure 1). In light of these facts it seems reasonable to assume that the mentioned massive demand of anemometers from this sector will continue in the coming years, if not increase.

Among the different instruments devoted to measuring wind speed, the cup anemometer is currently still the most used device in the wind energy sector [8], as it is inexpensive compared to alternative devices (e.g., sonic anemometers), it shows a linear response in the normal wind speed range [9] (as explained further in Section 3 of the present work, according to MEASNET procedures anemometer calibration wind speed ranges between 4 m·s^−1^ and 16 m·s^−1^ [10–12]), and is able to operate under quite extreme weather conditions [13].

**Figure 2.**
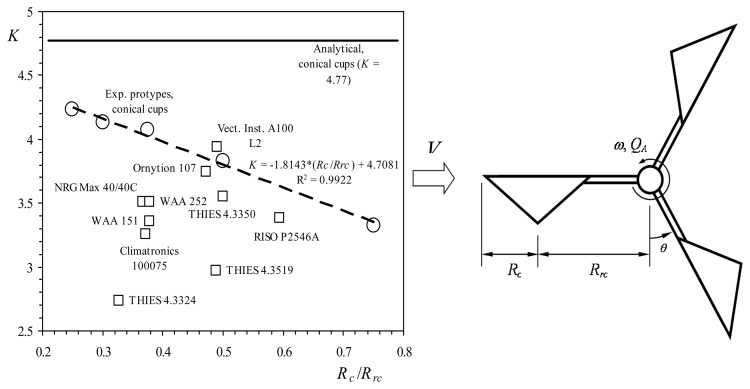
Cup anemometer factor, *K* (defined as *K* = *V*/2π*f_r_R_rc_*, where *V* is the wind speed, *f_r_* is the anemometer's rotation frequency, and *R_rc_* is the cups' center rotation radius), as a function of the ratio between the cups' radius, *R_c_*, and the cups' center rotation radius, *R_rc_* (see sketch on the right side). Experimental results (white circles) were measured with a Climatronics 100075 anemometer equipped with conical cup rotors built with the same cup radius, *R_c_* = 30 mm, and by varying the cups' center rotation radius, *R_rc_*. The linear fitting to these testing results has been added as a dashed line. The results from several commercial anemometers have been added to the graph, together with the analytical result calculated for the corresponding conical cups.

The cup anemometer was invented by Robinson in the 19th century [14–16]. It had four cups/arms instead of three, which is the present standardized configuration thanks to the work of Patterson [17], who found that the 3-cup anemometer is decidedly superior to the 4-cup due to a quicker and more uniform response, and a higher aerodynamic torque produced by the cups [18–20]. In 1924 the 3-cup anemometer was adopted as a standard for meteorology in the USA and Canada [20]. The behavior of this meteorological instrument has been widely studied throughout the twentieth century. Early studies focused on the optimal number of cups and arm length [21–23], cup aerodynamics [24–26], frequency recording system design [27–31], and errors due to fluctuating winds [20,24,32,33]. Following those initial efforts, researchers focused on cup anemometer response in turbulent flows, as the accuracy of wind speed measurements became increasingly important [9,34–48]. This research was applied to seek optimal calibration methods for these instruments, with special attention given to the impact of anemometer accuracy on the wind energy industry [11,12,49–56]. In addition, the importance of accuracy in wind speed measurements and the impracticality of constant recalibrations to maintain anemometer performance led researchers to study other aspects related to cup anemometer calibration. These include the impact of environmental (climatic) conditions [13,57–60], anemometer aging [61], the possibility of field calibration [62–64], the effects of wind stream non-uniformities, stream blockage and anemometer mounting arrangement on the calibration results [6,65–69], and uncertainties during the calibration processes [6,70]. Finally, recent efforts have been made to classify the different anemometers available on the market [70–75], and to compare the impact of shape on their performance [8,76–78]. The relationship between the performance of a cup anemometer and its shape has been studied experimentally, mainly through measurements of the aerodynamic normal-force coefficients on the cups, *c_N_*, as Breevort and Joyner [25] did in the past. Using those cup force coefficients as a function of the wind angle, some authors such as Schrenk [79], Wyngaard [41], Ramachandran [80,81], and Kondo [35] derived different analytical models to study cup anemometer behavior. Both analytical and experimental research on cup anemometer behavior has shown the correlation between anemometer transfer function (see Equation (1), below) and cup center rotation radius, *R_rc_*. However, some differences have also been highlighted between the results obtained using the two methods [76,77], see Figure 2.

**Figure 3.**
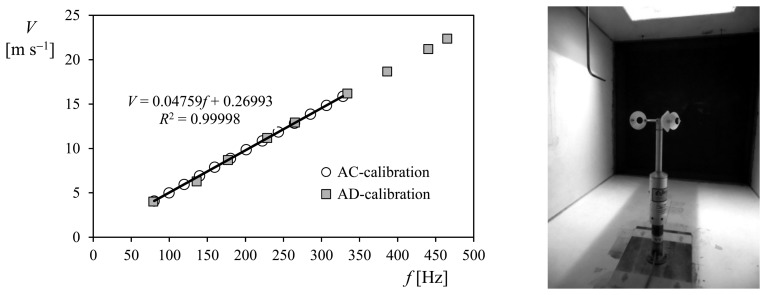
(Left) Results from two calibrations performed at the IDR/UPM Institute, on the same cup anemometer (Thies Clima 4.3350) following two different procedures, AC and AD. AC calibrations follow strictly MEASNET procedure (wind speeds ranging from 4 m·s^−1^ to 16 m·s^−1^, and 13 measurement points are taken), whereas AD calibrations are carried out over a broader wind speed range (from 4 m·s^−1^ to 23 m·s^−1^) and fewer measurement points are taken (9 instead of 13). The transfer function resulting from the linear fitting to AC calibration data has been included in the graph, together with the coefficient of determination, *R*^2^; (Right) Cup anemometer equipped with a prototype rotor during the calibration process at the S4 wind tunnel at the IDR/UPM Institute.

If, as aforementioned, the cup anemometer shows a linear behavior, then, the transfer function which relates the wind speed, *V*, to the output frequency of the anemometer, *f*, can logically be expressed by a linear equation:

(1) V=A⋅f+B

where A (slope) and B (offset) are calibration coefficients defined by means of a calibration process. In Figure 3, the results from two different calibrations performed on the same cup anemometer are shown. The transfer function corresponding to one of them has been included in the figure.

Looking for an equation with a clearer physical meaning, the transfer function can be rewritten in terms of the anemometer's rotation frequency, *f_r_*, instead of the output frequency, *f*:

(2) $$V=A_{r}\cdot f_{r}+B$$

where A*_r_* is the result of multiplying calibration constant A by the number of pulses per revolution given by the anemometer, *N_p_*. The number of pulses is different depending on the anemometer's inner system for translating the rotation into electric pulses. Magnet-based systems give 1 to 3 pulses per revolution, whereas optoelectronics-based systems normally give higher pulse rates per revolution, from 6 to 44 [8]. Finally, going back to the linearity of the cup anemometer performance on the aforementioned wind speed range, some authors claim that a non-linear equation should be used as the anemometer's transfer function instead of a linear one, especially at lower wind speeds [13,52]. Nevertheless, it should also be stated that the linear equation is used as, in normal working conditions, it is accurate enough and recommended in standard calibration processes [11,12,56,82].

## 2. Analytical Models to Study Cup Anemometer Performance

### 2.1. The 2-Cup Positions Analytical Model

To analyze the behavior of cup anemometer, analytical models have been proposed by other authors in the past. These models are developed from the following equation [56]:

(3) $$I\frac{\text{d}\omega }{\text{d}t}=Q_{A}+Q_{\text{f}}$$

where *I* is the moment of inertia of the rotor, *Q_A_* is the aerodynamic torque, and *Q*_f_ is the frictional torque, which depends on the air temperature, *T*, and the rotation speed, ω (from [73]: *Q*_f_ = *B*_0_(*T*) + *B*_1_(*T*)ω + *B*_2_(*T*)ω^2^, where coefficients *B*_0_, *B*_1_, and *B*_2_ are negative. Nevertheless, it should be also said that the friction torque, *Q*_f_, in Equation (3) has a negative sign in the updated version of reference [56]. Therefore, coefficients *B*_0_, *B*_1_ and *B*_2_ of the friction torque equation will be positive, if this is taken into account.

The frictional torque, *Q*_f_, can be neglected in the above equation, as it is normally very small in comparison to the aerodynamic torque [39,47], whereas the aerodynamic torque, *Q_A_*, can be derived from the aerodynamic forces on the rotor cups, which are normally measured in a wind tunnel in “static” configuration, that is, measuring the forces on an isolated and fixed cup immersed in a constant wind speed air flow and without considering any rotational speed (see Section 3 of the present work) [25,76,77]. In order to point out the reduced effect of the friction when compared to the aerodynamic forces, the work of Fabian [83] and Pedersen [73] should be mentioned. In Fabian's work the friction torque of a 3-cup anemometer (Riso P2244) was measured in the bracket [200 g·cm^2^·s^−2^, 500 g·cm^2^·s^−2^] for rotation speeds from 10 rad·s^−1^ to 55 rad·s^−1^. Following the formulae included in Pedersen's work, the friction torque of a similar anemometer (Riso P2546A) is calculated at 20 °C and 10 m/s wind speed as 2.1 × 10^−5^ N·m (same order of magnitude as the one measured by Fabian). If the aerodynamic forces are estimated in a cup of the last anemometer, the aerodynamic torque produced in one cup is around 1.6 × 10^−2^ N·m, that is, a much greater torque in comparison to the friction one.

**Figure 4.**
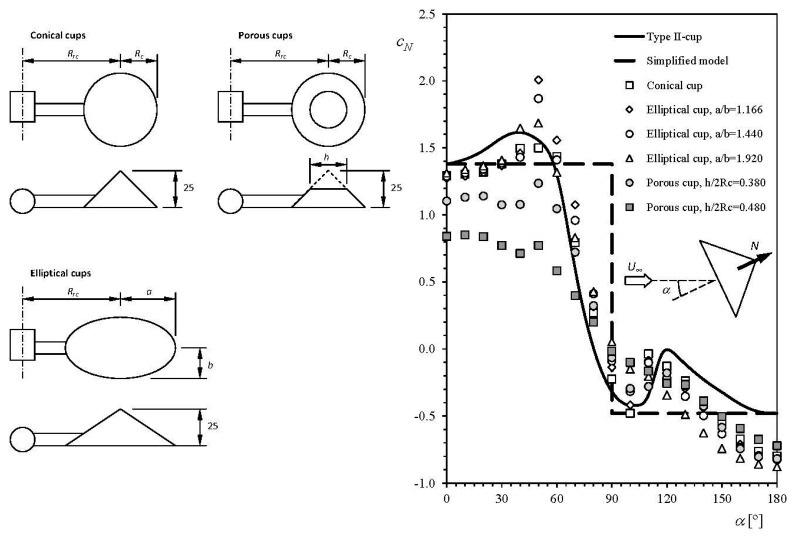
Normal-to-the-cup aerodynamic force coefficient, *c_N_*, regarding different cups: Brevoort & Joyner Type-II (conical) [25], conical, elliptical and porous [77] (see sketch on the left side, *R_c_* = 25 mm), plotted in relation to the wind direction with respect to the cup, α. The 2-cup analytical model simplification of the experimental results related to the Brevoort & Joyner Type-II cup, has been included as a dashed line.

Figure 4 shows the normal-to-the-cup aerodynamic force coefficient, *c_N_* (*c_N_* = *N*/0.5 ρ*V*^2^, where *N* is the normal-to-the-cup aerodynamic force, ρ the air density, and *V* the wind speed), measured on several anemometer cups of different types in “static” configuration (*i.e.*, not rotating), in relation to the wind angle with respect to the cup, α, [25]. This coefficient is normally introduced into Equation (3) to model the aerodynamic torque on the rotor, *Q_A_*, as in relation to the cups' aerodynamics. The 2-cup positions classical model (hereinafter, the 2-cup model) for aerodynamic torque is represented by the following equation:

(4) $$Q_{A}=\frac{1}{2}\rho S_{c}R_{rc}N_{c}\left[{\left(V-\omega R_{rc}\right)}^{2}c_{1}c_{D1}-{\left(V+\omega R_{rc}\right)}^{2}c_{2}c_{D2}\right]$$

where ρ is the air density, *N_c_* the number of cups, *S_c_* (*S_c_* = π*R_c_*^2^, where *R_c_* is the cup radius) the front area of the cups, *V* the wind speed, ω the rotational speed, *c_D_*_1_ (concave side) and *c_D_*_2_ (convex side) are the aerodynamic drag force coefficients of the cups, and *c*_1_ and *c*_2_ are coefficients that take into account the effectiveness of the aerodynamic simplification (for instance, rotating effects or even Reynolds number effects at low wind speed are not considered in the above equation), in which the aerodynamic torque is expressed as a function of the two more representative positions (in terms of aerodynamic force) of the cup in one turn. Normally, the effectiveness of the aerodynamic simplification is not considered in the equations, and it is assumed *c*_1_ = *c*_2_ = 1 [41,54,84]. The torque produced by each cup is then expressed as a function of the forces at only two positions, α = 0° (*c_D_*_1_ = |*c_N_*(0)|) and α = 180° (*c_D_*_2_ = |*c_N_*(180)|). Shown in Figure 4 is this simplification made on the results corresponding to the Type-II cup (dashed line). If the friction forces are left aside, as they are negligible in comparison to the aerodynamic ones [39,47], the above Equation (4) turns into:

(5) $$\frac{Q_{A}}{\frac{1}{2}\rho V^{2}\pi R_{c}^{2}R_{rc}(c_{D1}-c_{D2})N_{c}}=1-2\left(\frac{c_{D1}+c_{D2}}{c_{D1}-c_{D2}}\right)\left(\frac{\omega R_{rc}}{V}\right)+{\left(\frac{\omega R_{rc}}{V}\right)}^{2}$$

The above equation gives the stationary solution (*i.e.*, an averaged value of the rotation speed, ω), averaging it during one turn. So, as the average aerodynamic torque is equal to zero:

(6) $$0=1-2\left(\frac{c_{D1}+c_{D2}}{c_{D1}-c_{D2}}\right)\left(\frac{\omega R_{rc}}{V}\right)+{\left(\frac{\omega R_{rc}}{V}\right)}^{2}$$

and then, an equation for the anemometer factor, *K*, is obtained as a function of the aerodynamic characteristics of the cups (as aforementioned, characterized by coefficients *c_D_*_1_ and *c_D_*_2_):

(7) $$K=\frac{V}{\omega R_{rc}}=\frac{k_{d}+1}{k_{d}-1}$$

where:

(8) $$k_{d}=\sqrt{\frac{c_{D1}}{c_{D2}}}$$

In addition, this 2-cup model was successfully used in 1929 to analyze the overspeeding effect of the cup anemometer [41,79]. The cup anemometer overspeeding phenomenon consists of a quicker response upon wind flow acceleration than the one obtained after a wind flow deceleration. This causes an overrun of the cup anemometer (that is, an overestimation of the measured wind speed) in turbulent flows. This effect was already described in 1921 by Brazier [85,86]. More information on the overspeeding effect and Schrenk's work can be found in [84]. Additionally, it should also be pointed out that in a work by Kristensen and Lenschow [87], the mathematical description of the overspeeding states that it is affected by two different terms. The first one is produced by fluctuations with time scales shorter than the time constant of the anemometer, and it depends on the wind turbulence, whereas the second one depends on the anemometer transfer function curvature (*i.e.*, the calibration curve; MEASNET calibration procedures ensures a extremely reduced curvature is in the wind speed range from 4 m·s^−1^ to 16 m·s^−1^).

As said in the Introduction, the wind speed measurements done at present in meteorology or to control wind turbines are, despite the overspeeding effect, mainly carried out with Class-1 (Classification according to IEC 61400-12-1 standard [56].) cup anemometers (although sonic anemometers are increasingly used). It seems that, for the wind energy industry, accepting this slight deviation on the wind speed measurement by a mechanical instrument (the cup anemometer), is preferred than changing the procedures to include the performance of sonic anemometers, as these more modern instruments show higher levels of uncertainty and more complex calibration processes. It is also fair to say that the cup anemometer is a much mature instrument than the sonic anemometer, and therefore its physics have been more deeply analyzed. Additionally, it can be mentioned that at present MEASNET has not developed yet a specific calibration procedure for this kind of wind speed sensor. Going back to the overspeeding effect, it should also be mentioned that filtering techniques of the output signal can reduce this deviation [46,48,88,89]. However, as far as the authors' knowledge goes, this kind of technique have not being implemented in data loggers paired to cup anemometers installed in wind farms along Spain.

As previously said, cup anemometer overspeeding can be analyzed with the 2-cup model. Leaving aside the friction term, the following equation can be derived from Equations (3) and (4):

(9) $$\frac{I}{\frac{1}{2}\rho \pi R_{c}^{2}R_{rc}(c_{D1}-c_{D2})}\frac{\text{d}\omega }{\text{d}t}=V^{2}-2\phi V\omega R_{rc}+{\left(\omega R_{rc}\right)}^{2}$$

where:

(10) $$\phi =\frac{c_{D1}+c_{D2}}{c_{D1}-c_{D2}}$$

If small fluctuations of both the horizontal wind speed, *V*, and the rotational speed, ω, are considered (fluctuations only in the longitudinal direction of the wind are considered here in order to work with the simplest possible model):

(11) $$V=V_{0}(1+\nu \prime );\omega =\omega_{0}(1+\omega \prime )$$

then, Equation (9) can be rewritten as follows:

(12) $$\frac{I\omega_{0}}{\frac{1}{2}\rho V_{0}^{2}\pi R_{c}^{2}R_{rc}(c_{D1}-c_{D2})}\frac{\text{d}\omega \prime }{\text{d}t}=2\nu \prime +\nu^{\prime 2}-2\frac{\phi }{K}(\nu \prime +\omega \prime +\nu \prime \omega \prime )+\frac{1}{K^{2}}(2\omega \prime +\omega^{\prime 2})$$

where, as aforementioned, *K* is the anemometer factor (from Equation (7): *K* = *V*_0_/ω_0_*R_rc_* = (*k_d_* + 1)/(*k_d_* − 1)). The above equation can be rewritten as:

(13) $$\frac{I\omega_{0}}{\frac{1}{2}\rho V_{0}^{2}\pi R_{c}^{2}R_{rc}(c_{D1}-c_{D2})}\frac{\text{d}\omega \prime }{\text{d}t}+\frac{2}{K}\left(\phi -\frac{1}{K}\right)\omega \prime =\frac{2}{K}(K-\phi )\nu \prime +\nu^{\prime 2}+\frac{1}{K^{2}}\omega^{\prime 2}-2\frac{\phi }{K}\nu \prime \omega \prime$$

where:

(14) $$\phi -\frac{1}{K}=K-\phi =2\frac{k_{d}}{k_{d}^{2}-1}>0$$

and:

(15) $$\nu^{\prime 2}+\frac{1}{K^{2}}\omega^{\prime 2}-2\frac{\phi }{K}\nu \prime \omega \prime =(\nu \prime -\omega \prime )\left(\nu \prime -\frac{\omega \prime }{K^{2}}\right)$$

Then, the following equation can be obtained:

(16) $$\tau \frac{\text{d}\omega \prime }{\text{d}t}+\omega \prime =\nu \prime +R_{2}(\nu^{\prime 2};\omega^{\prime 2};\nu \prime \omega \prime )$$

where τ is the time constant (also called response time), which is defined as:

(17) $$\tau =\frac{I}{2\rho V_{0}\pi R_{c}^{2}R_{rc}^{2}\sqrt{c_{D1}c_{D2}}}$$

and *R*_2_ is a second order remainder term:

(18) $$R_{2}(\nu^{\prime 2};\omega^{\prime 2};\nu \prime \omega \prime )=\frac{{\left(k_{d}+1\right)}^{2}}{4k_{d}}(\nu \prime -\omega \prime )\left(\nu \prime -\frac{\omega \prime }{K^{2}}\right)$$

Equation (16) characterizes a first order linear time-invariant system [36,90], which fits to the real anemometer behavior well [82], and whose solution is well known:

(19) $$\omega \prime =\left(\nu \prime +R_{2}(\nu^{\prime 2};\omega^{\prime 2};\nu \prime \omega \prime )\right)(1-e^{-t/\tau })$$

In the above equations, it should be pointed out that, as the second order remainder term, *R*_2_, is always positive, as according to the aforementioned first order linear time-invariant systems, the response of the system, ω′, “follows” the perturbation *v*′ reaching 63% of its value at *t* = τ. Hence, a greater response of the anemometer when the incoming wind speed accelerates (positive values of *v*′) than when it decelerates (negative values of *v*′) is suggested, explaining the overspeeding phenomenon.

### 2.2. The 3-Cup Analytical Model

**Figure 5.**
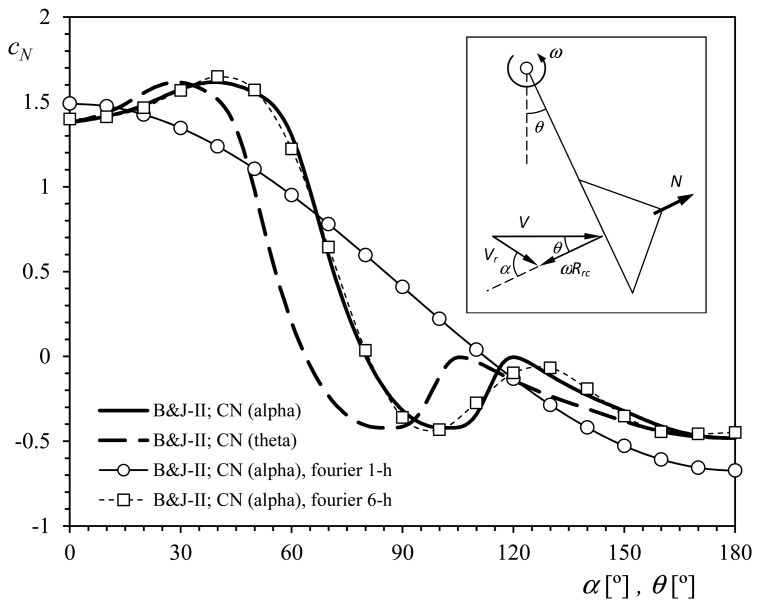
Experimentally measured normal aerodynamic force coefficient, *c_N_*, of the Brevoort & Joyner Type II (conical) cups [25], plotted as a function of the wind direction with respect to the cup, α. The coefficient is also plotted as a function of the rotor's rotation angle, θ (calculated with Equation (22) for an anemometer factor *K* = 3.5). See in the sketch the variables involved in the rotation of an anemometer's cup: normal aerodynamic force on the cup, *N*, wind speed, *V*, relative wind speed to the cup, *V_r_*, rotor's rotation angle, θ, rotor's rotational speed, ω, and wind direction with respect to the cup, α. The 1-harmonic term Fourier series approximation (Equation (24)) to the Type II cup has been plotted, together with the 6-harmonic terms Fourier series approximation (Equation (23)).

In order to approach the problem more accurately, the 3-cup analytical model was developed by integrating the aerodynamic normal force on all three cups in an entire rotation [35,76,81]. The starting point of this 3-cup model is the aforementioned Equation (3), taking into account, as stated, the aerodynamic torque produced by each cup of the anemometer rotor. If friction is also left out of Equation (3), the following equation can then be derived for the rotor movement:

(20) $$\begin{array}{l} I\frac{\text{d}\omega }{\text{d}t}=\frac{1}{2}\rho S_{c}R_{rc}V_{r}^{2}\left(\theta \right)c_{N}\left(\alpha \left(\theta \right)\right)+\frac{1}{2}\rho S_{c}R_{rc}V_{r}^{2}\left(\theta +120{}^{\circ}\right)c_{N}\left(\alpha \left(\theta +120{}^{\circ}\right)\right)+\\ +\frac{1}{2}\rho S_{c}R_{rc}V_{r}^{2}\left(\theta +240{}^{\circ}\right)c_{N}\left(\alpha \left(\theta +240{}^{\circ}\right)\right) \end{array}$$

where *V_r_* is the wind speed relative to the cups, *c_N_* is the aerodynamic normal force coefficient, α is the local wind direction with respect to the cups, θ is the angle of the rotor with respect to a reference line (see sketch in Figure 5), and *S_c_* is, as previously mentioned, the front area of the cups (*S_c_* = π*R_c_*^2^). Wind speed *V_r_*, relative to the cup at rotor angle θ with respect to the reference line, is expressed as:

(21) $$V_{r}(\theta )=\sqrt{V^{2}+{\left(\omega R_{rc}\right)}^{2}-2V\omega R_{rc}\cos(\theta )}$$

the wind direction with respect to the cup, α, being derived, as a function of the rotor's position angle, θ, from the following equation [76]:

(22) $$\tan(\alpha )=\frac{K\sin(\theta )}{K\cos(\theta )-1}$$

where *K* is the anemometer factor, defined by Equation (7).

Going back to Equation (19), it should be said that the experimentally measured normal aerodynamic force coefficient, *c_N_*, can be quite accurately simplified in terms of the Fourier series expansion [77]:

(23) $$c_{N}(\alpha )=c_{0}+c_{1}\cos(\alpha )+c_{2}\cos(2\alpha )+c_{3}\cos(3\alpha )+\ldots$$

See in Figure 5 the 6-harmonic term Fourier approximation to the Brevoort & Joyner Type II (conical) cups normal aerodynamic force coefficient [25]. A good agreement with the experimental results can be easily observed. Nevertheless, the first two coefficients can be considered enough to define a reasonable approximation to the experimental results:

(24) $$c_{N}(\alpha )=c_{0}+c_{1}\cos(\alpha )$$

if a comparison among the Fourier series coefficients is carried out. The above reduced approximation to the Brevoort & Joyner Type II (conical) cups normal aerodynamic force coefficient is also included in Figure 5.

Although this 1-harmonic-term equation does not seem to fit the wind tunnel results perfectly, the comparison among the different harmonic terms already carried out in a previous work [77] showed the much greater importance of the average and the first harmonic terms in relation to the other ones. Therefore, it could be assumed as a reasonable approximation for inclusion in Equation (19).

On the other hand, the related-to-the-cup wind angle, α, is quite accurately expressed in terms of rotor's rotation angle, θ, with the equation:

(25) $$\cos(\alpha )=\eta_{0}+\eta_{1}\cos(\theta )+\eta_{2}\cos{\left(\theta \right)}^{2}+\eta_{3}\cos{\left(\theta \right)}^{3}$$

where coefficients η_0_, η_1_, η_2_, and η_3_ can are expressed as a function of anemometer factor *K* (Equation (7)):

(26) $$\eta_{0}=\frac{-1}{\sqrt{1+K^{2}}};\eta_{1}=\frac{K}{\sqrt{1+K^{2}}}-\frac{1}{K^{2}-1};\eta_{2}=\frac{1}{\sqrt{1+K^{2}}};\eta_{3}=\frac{K^{2}}{K^{2}-1}-\frac{K}{\sqrt{1+K^{2}}}$$

Taking into account Equations (23)–(25), Equation (19) can solved for the stationary state by averaging the aerodynamic torque on one turn and making the result equal to zero. As a result, a direct relationship between the anemometer factor and coefficients *c*_0_ and *c*_1_ is obtained:

(27) $$0=\left(1+\frac{1}{K^{2}}\right)\left(1-\frac{1}{2}\frac{c_{1}}{c_{0}}\frac{1}{\sqrt{1+K^{2}}}\right)-\frac{1}{4}\frac{c_{1}}{c_{0}}\frac{1}{K}\left(\frac{1}{\sqrt{1+K^{2}}}+\frac{3K^{2}-4}{K^{2}-1}\right)$$

This equation gives the anemometer factor, *K*, as a function of the Fourier coefficients ratio *c*_1_/*c*_0_ (that only depends on the aerodynamics of the cup). Consequently, for each wind speed, *V*, the averaged value of the rotational speed of the rotor, ω, can be obtained from Equation (7). As in the case of the 2-cup model, an equation to analyze the overspeeding can be derived from the present model. If the non-harmonic part of Equation (19) is considered, it can be rewritten as:

(28) $$\frac{I}{\frac{3}{2}\rho S_{c}R_{rc}c_{0}}\frac{\text{d}\omega }{\text{d}t}=\left(V^{2}+{\left(\omega R_{rc}\right)}^{2}\right)\left(1+\frac{c_{1}}{c_{0}}\left(\eta_{0}+\frac{1}{2}\eta_{2}\right)\right)-\omega R_{rc}V\frac{c_{1}}{c_{0}}\left(\eta_{1}+\frac{3}{4}\eta_{3}\right)$$

then, if small fluctuations are considered Equation (11), the following equation for the first order terms can be derived after extracting the solution for the steady state (Equation (27)):

(29) $$\frac{I\omega_{0}}{3\rho V_{0}^{2}S_{c}R_{rc}c_{0}}\frac{\text{d}\omega \prime }{\text{d}t}=\nu \prime \left(k_{1}-\frac{k_{2}}{2K}\right)-\omega \prime \left(\frac{k_{2}}{2K}-\frac{k_{1}}{K^{2}}\right)$$

where *K* is the anemometer factor, *k*_1_ = 1+(η_0_+0.5η_2_)(*c*_1_/*c*_2_), and *k*_2_ = (η_1_+0.75η_3_)(*c*_1_/*c*_2_). Finally, Equation (29) can be simplified as:

(30) $$\tau \frac{\text{d}\omega \prime }{\text{d}t}+\omega \prime =\xi \nu \prime$$

which, as previously stated in the case of the 2-cup model, leads to the first order system solution:

(31) $$\omega \prime =\nu \prime \xi \left(1-e^{-t/\tau }\right)$$

where the time constant is defined as:

(32) $$\tau =\frac{I\omega_{0}}{3\rho V_{0}^{2}S_{c}R_{rc}c_{0}\left(\frac{k_{2}}{2K}-\frac{k_{1}}{K^{2}}\right)}$$

and:

(33) $$\xi =\frac{k_{1}-\frac{k_{2}}{2K}}{\frac{k_{2}}{2K}-\frac{k_{1}}{K^{2}}}$$

The above equations can be simplified using Equations (26) and (27) as:

(34) $$\tau =\frac{I}{\frac{3}{2}\rho V_{0}S_{c}R_{rc}^{2}c_{0}\left(K-\frac{1}{K}\right)\left(1-\frac{c_{1}}{c_{0}}\frac{1}{\sqrt{1+K^{2}}}\right)}$$

and:

(35) ξ=1

Reducing Equation (30) to the same equation derived using the 2-cup model:

(36) $$\omega \prime =\nu \prime \left(1-e^{-t/\tau }\right)$$

### 2.3. Accuracy and Limitations of Analytical Models

**Figure 6.**
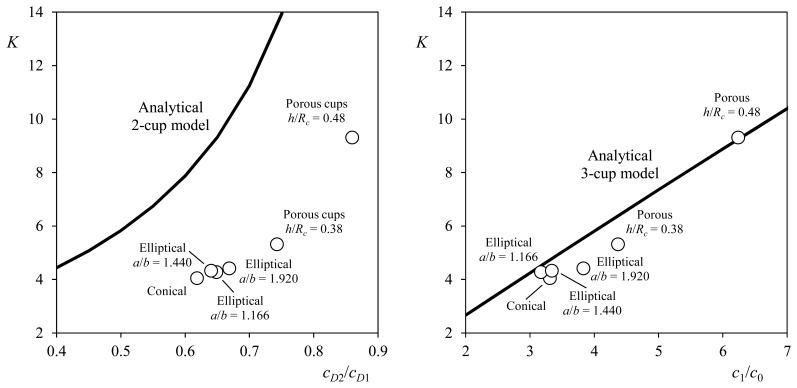
Comparison between results obtained with 2-cup (Left) and 3-cup (Right) analytical models and testing results. The anemometer factor is expressed as a function of the coefficients ratio *c_D_*_1_/*c_D_*_2_ (2-cup model) and *c*_1_/*c*_0_ (3-cup model). The experimental results correspond to calibrations performed on an Ornytion 107A anemometer equipped with different cup rotors. The cups shapes are sketched in Figure 4.

In Figure 6, the anemometer factor, *K*, calculated with the above explained analytical methods is shown, as a function, of the correspondent aerodynamic coefficient ratio. The results regarding calibrations performed on an anemometer equipped with different rotors (each one with different shape cups, see Figure 4) have also been included in the graphs. In this last case, the anemometer factor was calculated from the calibration coefficients of the transfer function (Equation (2)), leaving aside the offset, B:

(37) $$K=\frac{V}{\omega R_{rc}}=\frac{{\text{A}}_{r}f_{r}+\text{B}}{2\pi f_{r}R_{rc}}=\frac{{\text{A}}_{r}}{2\pi R_{rc}}\frac{1}{1-\left(\frac{\text{B}}{V}\right)}\approx \frac{{\text{A}}_{r}}{2\pi R_{rc}}$$

as this parameter has some effect limited to very low speeds [91].

The accuracy of both methods can be appreciated in the figure. Although the 2-cup method correctly reflects the cup aerodynamics effect on the rotor performance, it is clear that a more accurate solution is reached with the 3-cup method, as not only two cup positions are considered when calculating the aerodynamic torque on the rotor. However, it is also fair to say that the 2-cup model has shown itself to be a proper tool for analyzing the cup anemometer behavior, and not only with regard to the overspeeding effect. The 2-cup model has recently been used to study the pressure distribution on the anemometer rotation cups. The results being quite surprising as higher loads on the cups were found to be located close to the rotating axis, where the rotational speed is lower [91].

In terms of anemometer factor, *K*, the 3-cup model has a reasonable accuracy, around 10%, depending on the cup size [77]. In order to improve its accuracy the aforementioned non-constant pressure distribution on the cup surface should be considered. Nevertheless, new testing results are required in order to have an exact idea of the effect of rotation on the pressure distribution on the cups.

## 3. Testing Configuration and Experimental Setup

**Figure 7.**
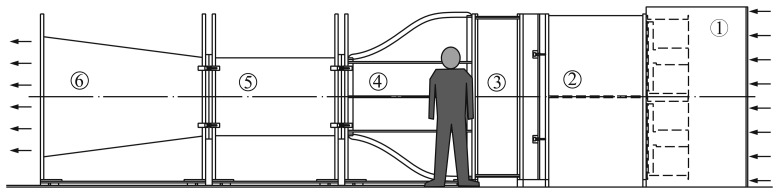
Sketch of the S4 wind tunnel of the IDR/UPM Institute devoted to anemometer calibration. (1). Fans; (2). Plenum chamber; (3). Honeycomb and grids; (4). Contraction; (5). Test chamber; (6). Diffuser.

The anemometer performance research done at the IDR/UPM Institute was based on anemometer calibrations, mainly performed in the S4 wind tunnel. This is an open-circuit wind tunnel served by four 7.5 kW fans, and with a 0.9 m × 0.9 m cross-section testing chamber (see Figure 7). The anemometer calibrations are performed in accordance with MEASNET requirements [11,12], which, in general overview, can be summarized as follows:

- The wind tunnel blockage ratio shall not exceed 0.05 for closed test sections.
- Turbulence intensity is limited to 0.02%.
- Flow quality measurement is carried out periodically.
- All transducers and measuring equipment have traceable calibrations.
- Prior to every calibration round, the integrity of the experimental set-up is verified.
- The repeatability of the calibration is verified periodically (target maximum difference between calibrations less than 0.5% at 10 m·s^−1^).
- Calibration are performed under both rising and falling wind speed in the range from 4 m·s^−1^ to 16 m·s^−1^, with 1 m·s^−1^ or less calibration interval.
- Anemometer calibration is supported by a detailed assessment of calibration uncertainty.

In addition to the above requirements, it should also be noted that IDR/UPM Institute is accredited in accordance with UNE EN-ISO/IEC 17025, as a calibration laboratory for fluid velocity measurements. Following requests from several customers in the past, the IDR/UPM Institute developed a different calibration procedure, consisting of a wider range from 4 m·s^−1^ to 23 m·s^−1^ covered with 9 measurement points instead of the 13 stated by the MEASNET procedures. See in Figure 3 the result of this reduced calibration (called AD calibration in the IDR/UPM internal procedures) compared to the MEASNET calibration (AC calibration).

**Figure 8.**
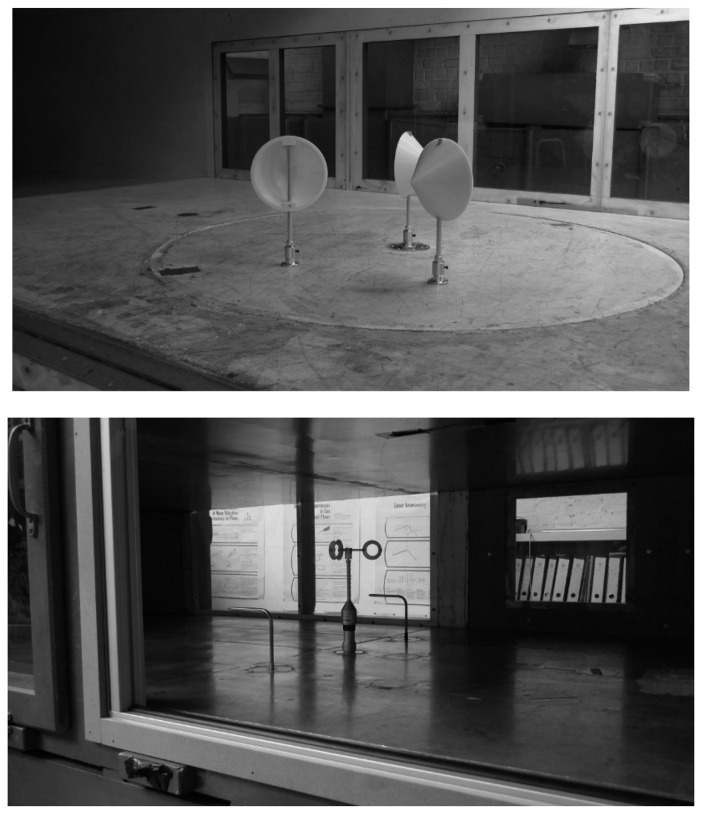
Wind tunnels at the Department of Mechanical Engineering at the Vrije Universiteit Brussel. (Top) Large wind tunnel for force measurements. The picture shows the aerodynamic force measurements on a cup in a “rotor” configuration (surrounded by two others); (Bottom) Anemometer calibration wind tunnel.

Some research campaigns planned by the IDR/UPM Institute were supported by the Department of Mechanical Engineering at the Vrije Universiteit Brussel, which has a solid reputation in the field of experimental and numerical aerodynamics. This department has two wind tunnels, one being used to measure the aerodynamic forces on cups (in “static” configuration, that is, non-rotating), and the second one being used for anemometer calibration (following the MEASNET procedure), see Figure 8. More information regarding both facilities can be found in [76,77].

## 4. Results and Discussion

### 4.1. Analysis of Calibration Results

Large series of commercial anemometers calibrated at the IDR/UPM were analyzed in a first attempt to extract some conclusions regarding its aerodynamic behavior [8]. The differences between AC and AD calibrations (see Section 3), in terms of changes on the Annual Energy Production (AEP) were also studied. Taking one of the IDR/UPM reference anemometer (Vector Instruments A100 L2), the AEP estimation error based on GE2.5 and Vestas V90 wind turbines and wind speed variation due to an AD calibration instead of a AC calibration, is around 0.6%–0.9% for low annual average wind speeds (4–5 m·s^−1^) and 0.2% for high annual average wind speeds (10–11 m·s^−1^). These errors are similar to the examples of uncertainties related to instruments and data acquisition systems included in the International Electrotechnical Commission (IEC) procedure [56]. This AEP estimation error was also calculated based on average results from different commercial anemometers (that is, comparing AEPs calculated with both AD and AC calibrations). Although the effect of enlarging the calibration wind speed range and reducing the number of points seems to be reduced (see Figure 3), some difference was observed when comparing, in terms of AEP, the results from First Class anemometers [71–74] to others from non-First Class anemometers, see Table 1, the First Class ones (RISØ P2546A; Thies 4.3350, Vector Inst. A100 L2) showing a more reduced variation of the AEP for lower annual average wind speeds at hub height.

**Figure 9.**
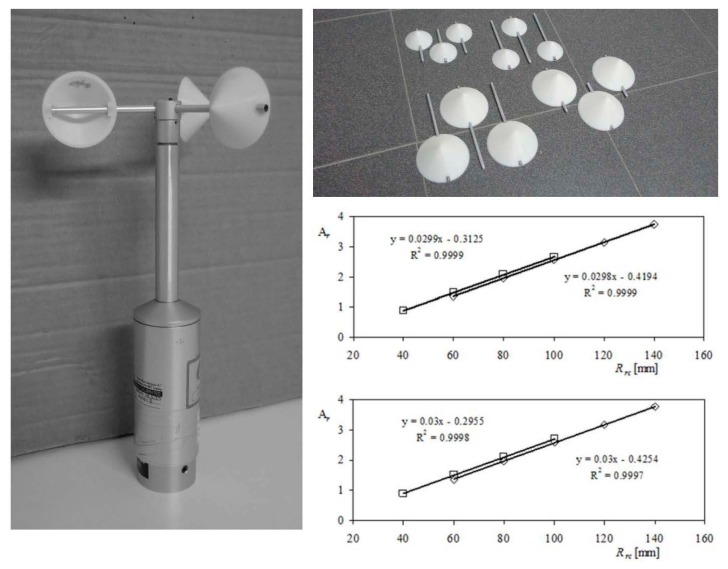
Climatronics 100075 anemometer equipped with conical cups rotor with dimensions: *R_c_* = 25 mm, and *R_rc_* = 60 mm (right). Some of the cups made of ABS plastic in a 3D printer (**top-right**); Calibration coefficients, A*_r_*, as a function of the cups' center rotation radius, *R_rc_* (**bottom-right**); Results from Climatronics 100075 (**top graph**); and Ornytion 107A (**bottom graph**) anemometers: squares stand for *R_c_* = 25 mm cups rotors, rhombi stand for *R_c_* = 40 mm cups rotors.

Another important conclusion derived from the aforementioned work [8] was the linear relationship between A*_r_* calibration constant and the cups center rotation radius, *R_rc_*. This particular effect was analyzed with a specific testing campaign whose results were included in a second work [76]. In that campaign two commercial anemometers (Climatronics 100075 and Ornytion 107A) were tested (*i.e.*, calibrated), equipped with 21 different conical-cup rotors (varying the cup radius, *R_c_*, and the cups center rotation radius, *R_rc_*). These rotors were formed with conical cups made in a 3D printer, and 5 mm diameter aluminum tube, see Figure 9.

After a thorough analysis of all calibrations data, the following equations were derived for the calibration coefficients of the anemometers transfer function (Equation (2)):

(38) $${\text{A}}_{r}=\frac{{\text{dA}}_{r}}{\text{d}R_{rc}}R_{rc}-S_{c}\left(\zeta +\eta S_{c}^{-\xi }\right)$$

(39) $$\text{B}=\left(\varepsilon +\phi S_{c}^{-\gamma }\right)R_{rc}-\mu S_{c}^{-\psi }$$

The most noteworthy conclusions reached in that research were:

- The slope of the calibration transfer function, A*_r_*, depends on two different contributions, one related to the cup center rotation radius, *R_rc_*, and the other related to the cups' front area, *S_c_*, or cup radius, *R_c_* (as *S_c_* = π*R_c_*^2^). The slope of Equation (38), dA*_r_*/d*R_rc_*, is related to the aerodynamic non-dimensional coefficient of the cups, as very small differences in this coefficient were observed among the 42 calibrations performed on the 2 anemometers tested. That is, the fitting coefficient dA*_r_*/d*R_rc_* did not seem to depend on the anemometer, with the same value for both the Climatronics 100075 and the Ornytion 107A anemometers, dA*_r_*/d*R_rc_* = 0.03 (with *R_rc_* expressed in mm, as indicated in graphs from Figure 9), whereas the other fitting coefficients, ζ, η, and ξ, were different depending on the anemometer tested.
- The offset of the calibration transfer function, B, also depends on the same shape parameters, although in this case each contribution is not totally independent of the next. In this case, all the fitting parameters, ε, γ, ϕ, μ and ψ, were different, depending on the anemometer tested.

Combining Equations (37) and (38), it is possible to derive a linear relationship between the anemometer factor, *K*, and the ratio of the cups to the cups center rotation radius (hereinafter denoted as *r_r_*; *i.e.*, *r_r_* = *R_c_*/*R_rc_*):

(40) $$K=\frac{1}{2\pi }\left[\frac{{\text{dA}}_{r}}{\text{d}R_{rc}}-\left(\zeta^{\prime }R_{c}+\frac{\eta \prime }{R_{2}^{2\xi -1}}\right)r_{r}\right]$$

The above linear relationship can be appreciated in Figure 2. Besides, another analysis of the experimental data from [76] was conducted in [91], the linearity of both A*_r_* and B coefficients being checked again.

**Figure 10.**
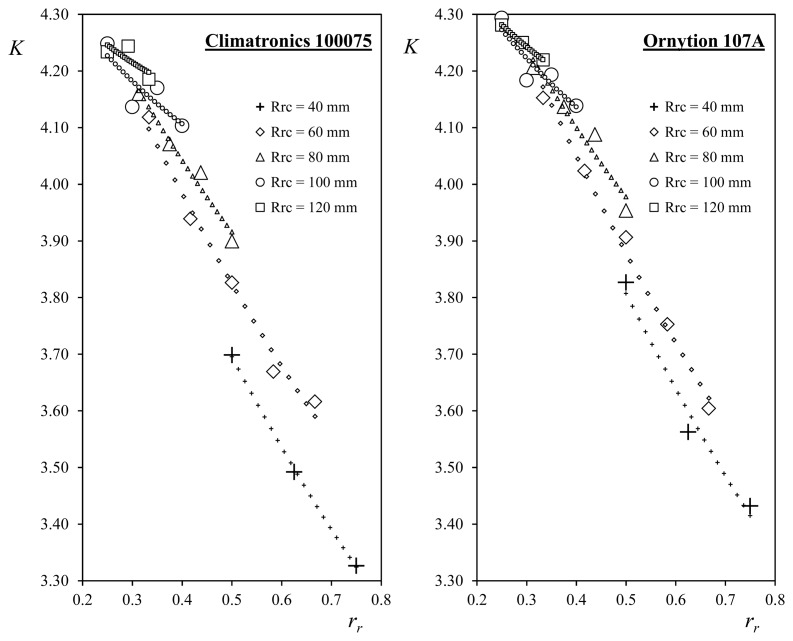
Anemometer constant, *K*, plotted as a function of the ratio *r_r_*, from multiple calibrations performed on Climatronics 100075 and Ornytion 107A cup anemometers, equipped with different conical-cup rotors (see picture in Figure 9). Results of the 2-cup analytical model implemented with non-constant aerodynamic load distribution on the cups are also included in the plots (small symbols).

As previously mentioned in Section 2.3, in this more recent work the results were analyzed in comparison with the 2-cup analytical model. As a result, it was revealed that the only way to fit the analytical model to the experimental data was considering a non-constant force distribution on the cups, with the highest load being located in the area of the cups closer to the rotation axis (which is where the cups' speed due to the rotational movement is lower). In Figure 10 the results of this work are included. The anemometer constant, *K*, is plotted as a function of the ratio *r_r_*, the data being derived from the multiple calibrations performed on Climatronics 100075 and Ornytion 107A cup anemometers, equipped with different conical-cup rotors (see picture in Figure 9). In the plots included in the aforementioned figure, the linear relationship between the anemometer factor, *K*, and the ratio *r_r_* can be observed, together with the effect of the cups' radius, *R_c_*, which affects the slope of the linear behavior observed. Besides, the results of the 2-cup analytical model with the mentioned non-constant aerodynamic load distribution on the cups implemented are also included in the plots (small symbols). It can be appreciated that a good match with the testing results is showed by the analytical model.

### 4.2. Variation of Calibration Results with Air Density

The influence of the environmental conditions during the calibration process on the cup anemometer transfer function (*i.e.*, the calibration constants A and B), was analyzed in 2012 at the IDR/UPM Institute [57]. Ambient conditions, especially changes in air density from the value at sea level (ρ = 1.225 kg·m^−3^), are taken into account in the IEC 61400-1 International Standard [92] with regard to the wind mills power curve measurement and AEP estimations. More specifically and in relation to the anemometers' behavior, in the IEC 61400-12-1 International Standard [56] the air density, ranging from ρ = 0.9 kg·m^−3^ to ρ = 1.3 kg·m^−3^, is defined as an influence parameter for anemometer classification. The importance of taking into account changes in air density must be underlined, as wind energy production estimations depend linearly on this parameter.

Regarding cup anemometer calibration, the effect of density changes on cup anemometer performances has been reported in the past [93]. In a classical work by Schubauer and Mason [94] the changes of density on anemometer calibration is experimentally studied using air and water as working fluid. Also, an interesting dimensional analysis is included in this reference. As a result, these authors suggest an equation to take into account changes in the working fluid (*i.e.*, the air density):

(41) $$V_{0}\sqrt{\rho_{0}}=V\sqrt{\rho }$$

where *V*_0_ and ρ_0_ are respectively the velocity and air density from the calibration process, and *V* the expected velocity ρ air density.

**Figure 11.**
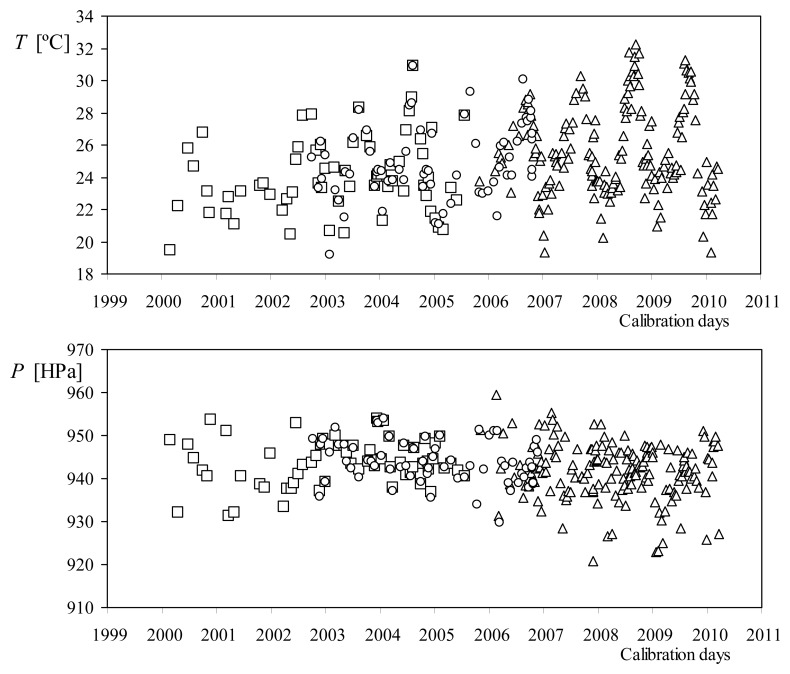

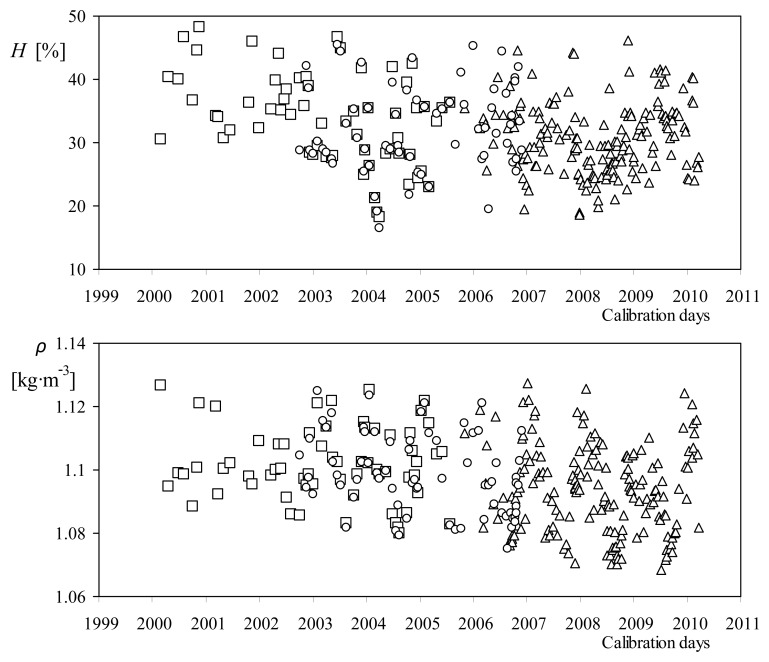
Climatic conditions and air density during calibrations performed on the anemometers used at the IDR/UPM Institute for quality control procedures: Climatronics 100,075 (squares), Vector instruments A100 L2 (circles), and Thies Clima 4.3350 (triangles), from January 2001 to February 2010. From [57].

Logically, the air density at the IDR/UPM Institute changes depending on the climatic conditions, and especially on temperature (see Figure 11).

**Figure 12.**
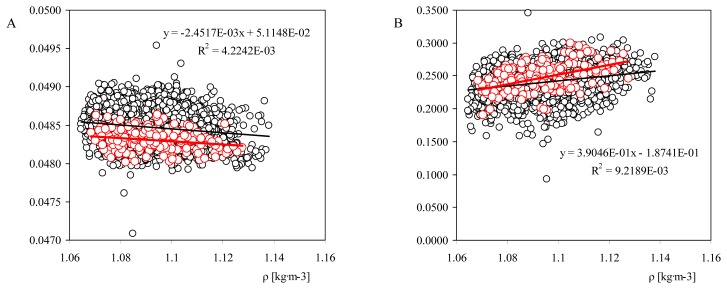
Calibration constants, (A, B, see Equation (1)), measured for the Thies Clima 4.3350 model anemometers (new anemometers, *i.e.*, first calibration), as a function of the air density value, ρ, during the calibration process. The data corresponding to the calibrations performed for quality assurance processes on the IDR/UPM Institute Thies Clima 4.3350 anemometer have been also included (red color). The linear fits to both data sets have been also included in the graphs. From [57].

These changes on the climatic conditions are reflected on the calibration constants of the anemometer, as suggested by the classical theory. In Figure 12, the transfer function constants, A and B, from calibrations performed on new Thies Clima 4.3350 model anemometers are shown, in relation to the average air density, ρ, during the calibration process.

The data corresponding to the Thies Clima 4.3350 anemometer used for internal procedures at the IDR/UPM Institute have been also included in the graphs. A quite scattered behavior is observed in the figure, although the linear trend of A and B constants is also clear. This trend has suggested a quite high impact on the Annual Energy Production (AEP) estimations [57]. More research should be done in this particular matter, as in the study carried out the air density variations are mainly driven by changes in temperature, which can have a significant effect on the frictional torque (see Equation (3)), especially at low temperatures [72,75]. This friction torque has been found to have influence in both calibration constants, A and B [95,96]. As a result, it should be underlined that even if some effect of air density should be expected on the cup anemometer performances, as it changes the ration between aerodynamic and friction toques, it is also quite difficult to filter this effect from changes produced by temperature variations.

### 4.3. Effect of Aging on Cup Anemometer Performances

Together with the analyses of air density variations effect on the cup anemometer performances, a specific research study was carried out in 2012 regarding the loss of performance due to aging. It seems reasonable to assume that once an anemometer is in service, the loss of performance should modify both calibration constants, A and B.

On the one hand, this degradation due to wear and tear could affect the anemometers' rotational speed, that is, the anemometers' capacity to transform energy from wind into rotation of the shaft should be reduced if energy losses increase (friction, for example), or the rotor's moment of inertia or its aerodynamics are changed by the mass addition of dirt. The reduction in the rotational speed can be translated into an increase of the constant A value. On the other hand, the degradation could also affect the starting speed of the anemometer, that is, as it is longer in service the wind speed necessary to start its rotation could be higher if the friction has increased, and that effect can be translated into an increase of the constant B. Together with the aforementioned considerations, it should also be noted that the anemometer's rotor could have a transitional period of time at the beginning of its life service before reaching its stable working condition, as common in complex mechanisms.

Two different anemometers' degradation cases were analyzed. The first one is the degradation of anemometers not used in field and just stored. This case was studied with the data from many calibrations performed on three different single individual anemometers only used to test the calibration wind tunnel. These calibrations are periodically carried out as part of the internal quality control procedures at the IDR/UPM Institute, with no maintenance programmed for these anemometers. The second case is related to the degradation of anemometers used in the field. The data from calibrations performed on the same anemometers, sent several times to the IDR/UPM Institute, were collected and analyzed in order to study the degradation of five different models of anemometers. Five enterprises of the wind energy sector (Barlovento, Cener, Dekra Ambio, Ecosem, and Ges-Siemsa) worked together with the IDR/UPM Institute in order to complete the information and strengthen the study with regard to the anemometers' behavior once in service. Thanks to the information provided by the aforementioned enterprises, the maintenance work on several individual anemometers was traced. Some of these anemometers were subjected to high level maintenance, normally consisting of changing the bearings (sometimes together with the change of the anemometer's electronics and the cups' rotor, if damaged).

**Figure 13.**
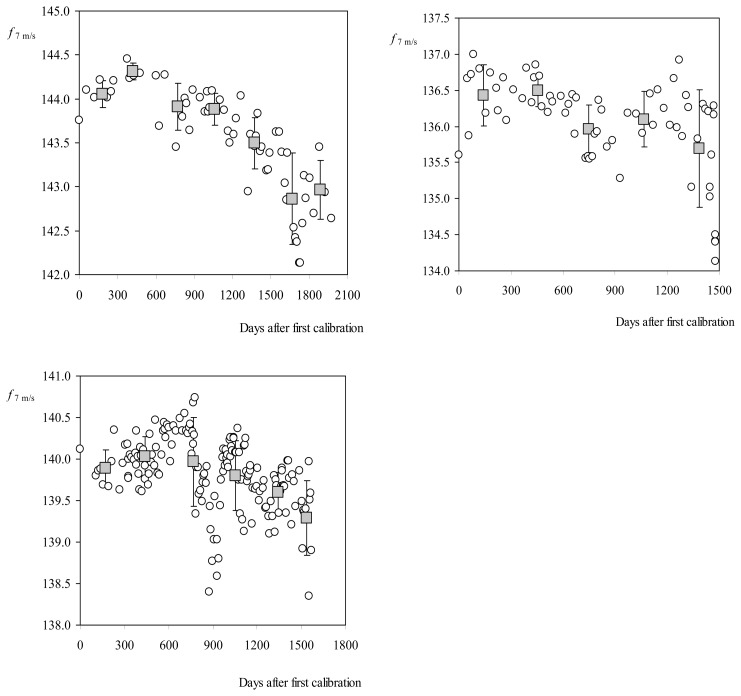
Evolution of the output frequency at 7 m·s^−1^ wind speed, *f*_7 m/s_, as after the first calibration, regarding the reference anemometers for quality assurance processes at the IDR/UPM Institute: Climatronics 100075 (**Top left side**); Vector Instruments A100 L2 (**Top right side**); and Thies Clima 4.3350 (**Bottom**). Obviously, these anemometers were not used in the field and, therefore, they are not considered to be affected by wear and tear. The 300-day average value has been included (grey squares), together with the standard deviation bars.

**Figure 14.**
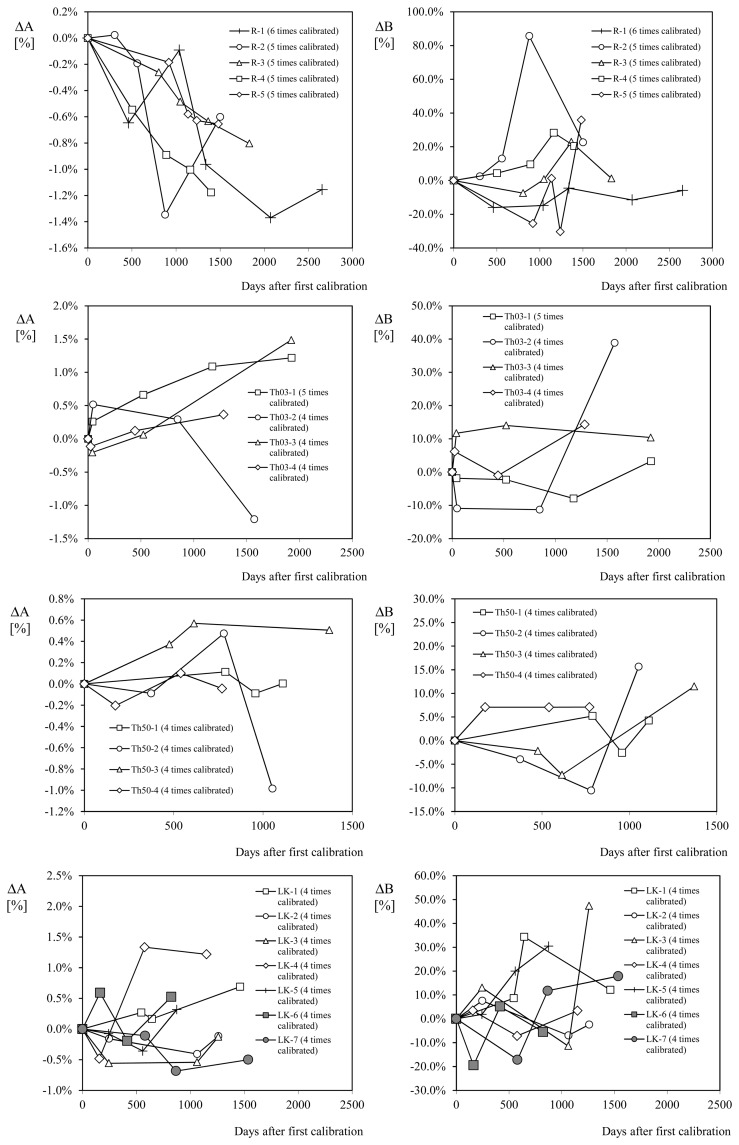
Percentage variation of calibration constants A and B from the initial values with regard to (from top to bottom): Risø P2546 anemometers (R-1 to R-5) calibrated more than four times; Thies Clima 4.3303 anemometers (Th03-1 to Th03-4) calibrated more than three times; Thies Clima 4.3350 anemometers (Th50-1 to Th50-4) calibrated more than three times; and Vector Instruments A100 LK anemometers (LK-1 to LK-7) calibrated more than three times. See in Table 2 the maintenance procedures on these anemometers between calibrations.

After this study two main conclusions arose. In new and not used anemometers, there is a transitional period in which the cup anemometer performance is adjusted. At the beginning of this period the performances are increased, that is, the rotation frequency grows until a maximum peak and then the performance is degraded by the normal wear and tear (see Figure 13). The second conclusion being that the task of estimating the level of degradation regarding anemometers operation on the field is subjected to a large uncertainty, if these estimations are based on variations of the transfer function constants. In Figure 14, the percentage variation of the transfer function constants A and B in relation to some anemometers calibrated several times at the IDR/UPM Institute, is plotted as a function of the number of days elapsed since the first calibration. Some of these anemometers had maintenance after the service period, and before their calibration at the IDR/UPM wind tunnel (see Table 2).

### 4.4. Anomaly Detection in Damaged Cup Anemometers

As previously mentioned, it is a fact that cup anemometers show degraded performance due to the normal wear and tear process. Besides, snow, rain and other climatic phenomena can seriously compromise the integrity of the anemometer, and dirt accumulation can modify both the aerodynamics of the rotor and its moment of inertia (and also, the correct balance of the bearings system). It should also be mentioned that as around 30% of mast-mounted anemometers return for recalibration far from normal operational conditions [97].

Until now, cup anemometer working condition had to be checked through frequent calibrations [63]. In addition, Calibration-on-the-field procedures have been studied as a cost-effective solution in order to monitor the cup anemometers status, and simplify their maintenance [62,64]. As a result of the concerns of the industry on this matter several patents and inventions have been developed [98–106]. Furthermore, some interesting results in relation to cup anemometers working condition status were achieved as a result of the PHM 2011 Data Challenge Competition. In that challenge, a set of measurements (mean, standard deviation, maximum and minimum wind speed), taken by several paired anemometers installed at different heights along a vertical mast were analyzed in order to study the anemometers' working condition. Different solutions were obtained by the researchers who took part in that challenge, the most significant ones being based on: Direct comparison of signals from two different cup anemometers, once properly filtered [107]; the correlation of the differences in measured wind speed from two anemometers with a Weibull distribution [108]; and the use of a neural network model [109]. The neural network model approach seems to be a quite accurate tool for cup anemometer performance analysis, as it obtained the highest score in the PHM 2011 Data Challenge Competition, and it has also been used with good results to compensate anemometer overspeeding in real-time measurements [89].

At present, a new way to monitor the cup anemometer status is being developed at the IDR/UPM Institute. It is based on the Fourier analysis of the rotation speed during one turn of the rotor [78]. Due to its 3-cup standardized configuration rotational speed of the cup anemometer is not uniform [75], and consequently, under a perfectly constant and uniform wind speed the rotational speed can be decomposed along one turn into a constant term, ω_0_, and a series of harmonic terms that correspond to a frequency three times bigger than the one related to the mentioned constant term, 3ω_0_, and its multiples, 6ω_0_, 9ω_0_, 12ω_0_…:

(42) $$\omega (t)=\omega_{0}+\sum_{n=1}^{\infty }\omega_{3n}\sin(3n\omega_{0}t+\varphi_{3n})$$

**Figure 15.**
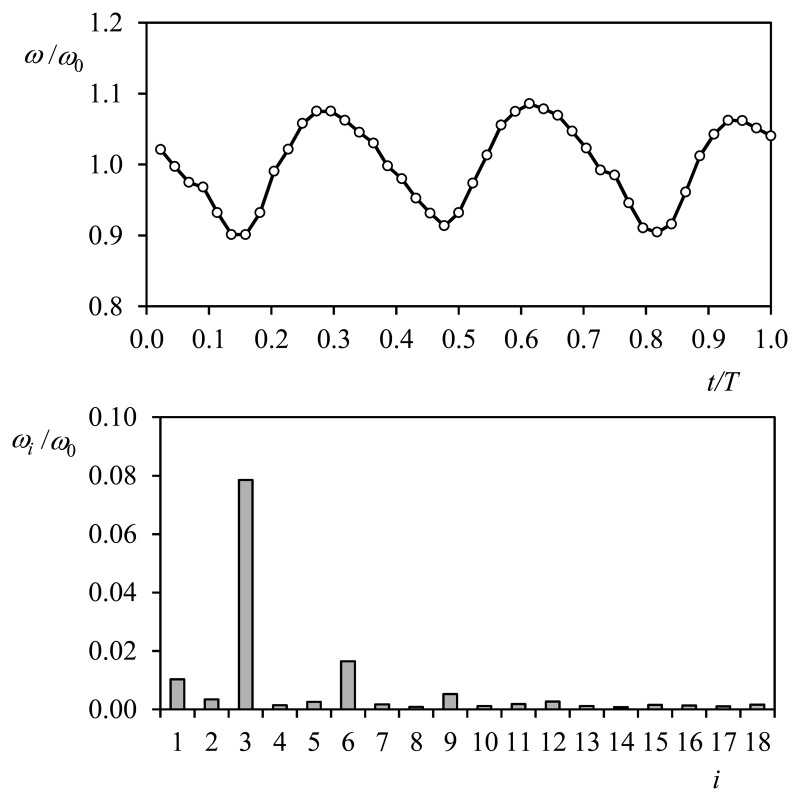
Relative-to-the-average rotational speed, ω/ω_0_, of a Thies 4.3303 anemometer during one turn at 8 m/s wind speed [75] (**Top**); and non-dimensional values of the Fourier series decomposition performed on that rotational speed, ω*_i_*/ω_0_ (**Bottom**), see Equation (42). *T* is the period of the anemometer's rotation.

In the top graph of Figure 15, the non-dimensional rotation speed, ω(t)/ω_0_, of a Thies 4.3303 cup anemometer at 8 m/s wind flow is shown as a function of time during one turn. The harmonic terms corresponding to the Fourier series decomposition of that signal (*i.e.*, the rotational speed) are included in the bottom graph of the aforementioned figure. In the bottom graph, the greater influence of the third harmonic term, ω_3_/ω_0_, which is clearly noted in the top graph, is mathematically confirmed.

The harmonic terms of several cup anemometers equipped with different rotors have been calculated during their calibrations performed as described in Section 3. The output signal was recorded taking 10,000 samples during 20 s for each calibration wind speed (that is, 13 records in each calibration). From each record the Fourier decomposition was performed on the data resulting from averaging several revolutions. Finally, the harmonic terms were obtained averaging the corresponding ones from the 13 records taken at the calibration wind speeds:

(43) $${\bar{\omega }}_{i}=\frac{1}{13}{\sum_{j=1}^{13}\frac{\omega_{i}}{\omega_{0}}|}_{j}$$

**Figure 16.**
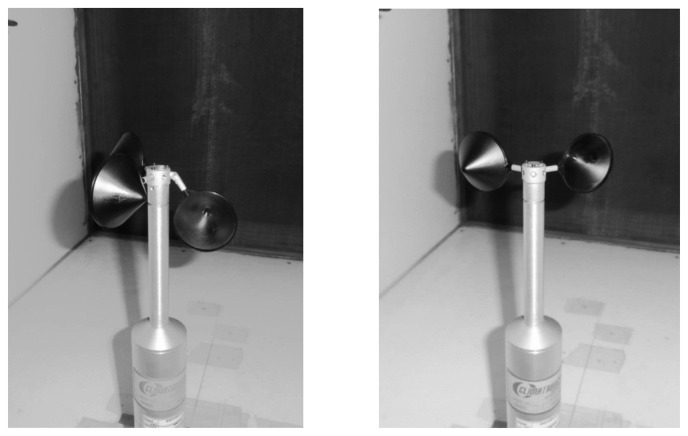
Climatronics 100075 cup anemometer equipped with two damaged rotors: Damaged cup-arm rotor (**Left**) and 1-missing-cup rotor (**Right**).

**Figure 17.**
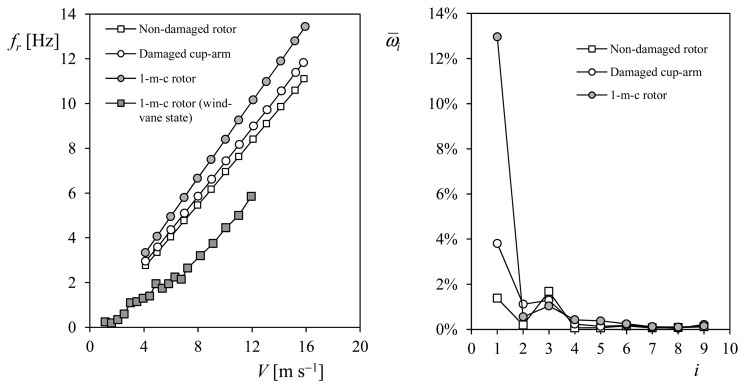
Rotational frequency, *f_r_*, in relation to the wind speed during calibration, *V*, (**Left**); and non-dimensional averaged harmonic terms, *ω̅_i_*, corresponding to a Climatronics 100075 cup anemometer equipped with non-damaged, damaged, and 1-missing-cup (1-m-c) rotors (**Right**). The rotation frequency based on the output voltage signal of the anemometer equipped with the 1-m-c rotor in the wind-vane equilibrium state is also included in the left graph. See in Figure 15 pictures of the damaged cup-arm and 1-missing-cup rotors).

In Figure 16 the pictures of two damaged rotors mounted on a Climatronics 100075 cup anemometer are included. On the left picture, a rotor with a damaged cup-arm is shown, whereas on the right the rotor has only two cups (this configuration will be referred hereinafter as 1-missing-cup rotor, *i.e.*, 1-m-c rotor). The rotation frequency of the aforementioned anemometer equipped with the damaged rotors is plotted in relation to the wind speed, in the left graph of Figure 17. Also, the rotation frequency of the anemometer equipped with a non-damaged rotor in included in the graph. These data were obtained from valid calibrations taking into account MEASNET requirements (see Section 3), that is, the regression coefficient was above 0.99999 in all three cases. As can be observed in the graph, the non-damaged rotor seems to show a worse aerodynamic efficiency than the damaged rotors, with lower rotational speed for the same wind speeds. In accordance with the classical theory of rigid-body dynamics, the explanation for this behavior lies in the lower values of the moment of inertia regarding the damaged anemometers (in the first case one cup is closer to the rotation axis whereas in the second one the lack of one cup directly involves a reduction in the moment of inertia), although the aerodynamic forces are reduced in the damaged rotors when compared to the non-damaged one.

Then, not only can transfer functions of cup anemometers equipped with damaged rotors be as linear as the ones from non-damaged cup-anemometers, but also these damaged instruments can rotate at higher frequencies. These effects result in two major drawbacks. For one thing, the wind speed measured by one of these damaged anemometers operating on the field would be incorrect, and for another, it would be quite difficult to detect this problem by only reviewing the wind speed measurements.

As aforementioned, the anemometer calibration team at the IDR/UPM Institute is working on a solution based on filtering the output signal, and extracting the harmonic terms corresponding to the rotational movement of the rotor. Any anomaly (dirt, damage…) introduced on the anemometer should affect the first harmonic term, *ω̅_1_*, as it would cause a perturbation which will be repeated once per turn. In Figure 17 the above explained Fourier series decomposition calculated with the anemometer output signal record during the calibrations considered is shown. As can be appreciated in the figure, the anemometer equipped with damaged rotors presents higher values of the first harmonic term when compared to the case equipped with the non-damaged rotor, revealing the anomaly present in each damaged rotor.

**Figure 18.**
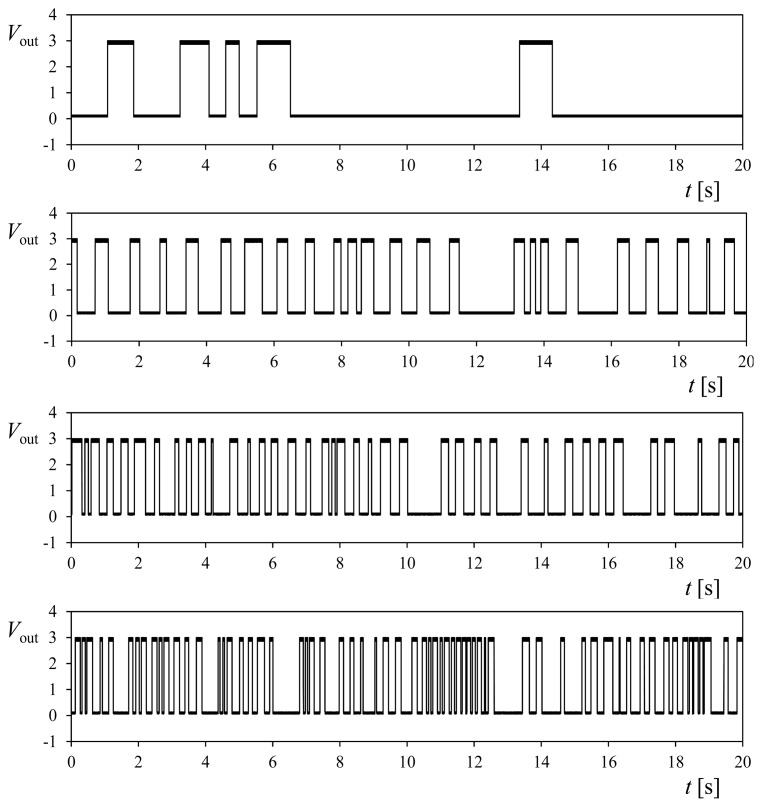
Voltage output, *V*_out_, of Climatronics 100075 anemometer equipped with the 1-missing-cup damaged rotor at stabilized position. Sampling during 20 s at 10,000 Hz, for (from Top to Bottom) *V* = 1.13, 3.48, 5.83 and 8.18 m·s^−1^ wind speed.

Besides, it should also be noted that a damaged anemometer can give false information to the measuring system if the symmetry of the rotor is affected. In certain cases, the asymmetry of the rotor can make it to behave like a wind vane. The 1-missing-cup damaged rotor (see Figure 16), was found to have a stable-equilibrium position at all wind speeds tested. Nevertheless, the anemometer produced several pulses, as some tiny oscillations of the rotor around the stable position were produced by the wake downstream the anemometer's “neck”. These oscillations of the rotor are transmitted through the shaft to the 30-hole perforated disk, which is responsible for the pulse generation at the opto-electronic system of the Climatronics 100075 anemometer. In Figure 18 the output signal, *V*_out_, from the anemometer equipped with the 1-missing-cup damaged rotor, recorded at 1.13, 3.48, 5.83 and 8.18 m·s^−1^ wind speeds is shown. These signals can be misinterpreted by the measurement system, giving then a false measurement of the wind. See in Figure 17 the “false” wind speed measurements if the 2-cup damaged rotor is stabilized at the equilibrium point.

Finally, the position of the 1-missing-cup damaged rotor in wind-vane state, that is, in the stabilized equilibrium position can be estimated with the 3-cup analytical model. From Equation (20), and taking into account the static state of the rotor (ω = 0; and α = θ):

(44) $$0=\frac{1}{2}\rho S_{c}R_{rc}V^{2}c_{N}\left(\theta \right)+\frac{1}{2}\rho S_{c}R_{rc}V^{2}c_{N}\left(\theta +120{}^{\circ}\right)$$

then:

(45) $$0=c_{N}\left(\theta \right)+c_{N}\left(\theta +120{}^{\circ}\right)$$

which, taking into account the simplification proposed (Equation (23)), can be rewritten as:

(46) $$\sin(\theta -30{}^{\circ})=\cos\left(\theta -120{}^{\circ}\right)=2\frac{c_{0}}{c_{1}}$$

**Figure 19.**
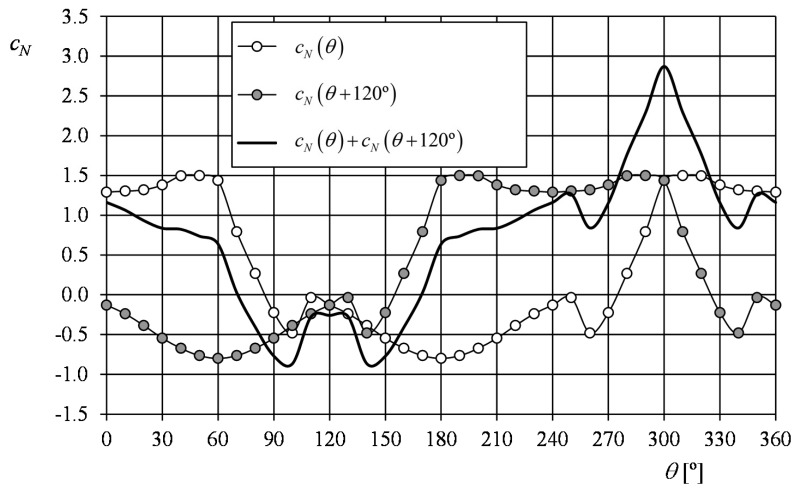
Normal-to-the-cup aerodynamic cup coefficients, *c_N_*(*θ*) and *c_N_*(*θ* + 120°), corresponding to each one of the two cups of the 1-missing-cup damaged rotor (left picture in Figure 16), in relation to the rotor's position. The addition of both coefficients is also included in the graph.

The solution of the above equation can be obtained, for the conical cups of Figure 4, taking into account its coefficients ratio *c*_1_/*c*_0_ = 3.312 from [77]. In fact, two solutions are obtained, θ = 67° and θ = 172°. In Figure 19, the aerodynamic coefficients of both cups of the rotor (from Figure 4), are plotted in relation to the first cup angular position, θ. The sum of both coefficients has been added to the figure. This last graph indicates θ = 71° and θ = 169° as solutions for Equation (45).

## 5. Conclusions/Outlook

Since 2008 several research works have been carried out at the IDR/UPM Institute regarding cup anemometer performances (*i.e.*, the transfer function). The most noteworthy conclusions from these works are:

- Analytical models, 2-cup and 3-cup, are a proper mathematical tool for studying particular effects on the cup anemometer performance. The 3-cup analytical model represents an improvement in terms of accuracy when compared to the 2-cup analytical model. However, it also presents a higher degree of complexity.
- A reduced calibration in terms of points (9 instead of 13) and within a wider wind speed range (from 4 m·s^−1^ to 23 m·s^−1^), compared to the one required by MEASNET procedures, has a reduced impact in relation to the measured wind speed and, more important, in terms of Annual Energy Production (AEP) estimations. Calculated AEP of a GE2.5 wind turbine showed differences of 0.67%–0.77% (4 m·s^−1^ average sped at hub height), 0.32%–0.34% (7 m·s^−1^ average sped at hub height), and 0.18%–0.19% (10 m·s^−1^ average sped at hub height), depending on the calibration procedure (Class-1 anemometers).
- The rotor's geometry of cups anemometers has a great impact on the calibration constants of the anemometer's transfer function. The most significant parameter being the ratio of the cups, *R_c_*, to the cups center rotation radius, *R_rc_*, that is, *r_r_* = *R_c_*/*R_rc_*. Besides, the cups area and the moment of inertia can have some coupled effect on the anemometer performances. This particular is currently being experimentally studied at the IDR/UPM Institute.
- The effect of climatic changes on the anemometer performance can be measured. Nevertheless, it is quite difficult to detach the temperature changes effect from the effect of changes on the air density, as changes on temperature affect both the air density and the frictional torque.
- Despite the difficulties of analyzing the effect of ageing on the anemometers, a new methodology that is currently under development at the IDR/UPM Institute to detect anomalies could be used to estimate the degree of wear and tear. This methodology, based on the Fourier series decomposition of the cup anemometer's output signal, has been successfully used to detect different levels of damage on rotors.

## Figures and Tables

**Table 1.** Percentage variation in the Annual Energy Production (AEP) of a GE2.5 wind turbine, related to the differences in measured wind speed between AC and AD calibrations [8].

**Cup Anemometer**	**Annual Average Wind Speed at Wind Mill Hub Height**		

	**4 m·s^−1^**	**7 m·s^−1^**	**10 m·s^−1^**
NRG Maximum 40/40C	0.91%	0.45%	0.25%
NRG IceFree	4.53%	1.99%	1.07%
RISØ P2546A	0.67%	0.32%	0.18%
Thies 4.3350	0.75%	0.34%	0.19%
Thies 4.3303	1.29%	0.42%	0.21%
Thies 4.3520	0.82%	0.39%	0.22%
Vector Inst. A100 L2	0.68%	0.32%	0.18%
Vector Inst. A100 K	0.77%	0.34%	0.19%
Ornytion 107	1.75%	0.72%	0.38%
RM Young 3002	2.12%	0.72%	0.37%

**Table 2.** Maintenance works performed on anemometers calibrated several times at the IDR/UPM Institute. See also Figure 14.

**Risø P2546**					

**Anemometer**	**Maintenance before Calibration**				

	**2nd**	**3rd**	**4th**	**5th**	**6th**
R-1	No	No	No	Yes °	No
R-2	No	No	No	No	-
R-3	Yes °	No	No	No	-
R-4	No	No	No	No	-
R-5	Yes °	No	No	No	-

**Thies Clima 4.3303**					

**Anemometer**	**Maintenance before Calibration**				

	**2nd**	**3rd**	**4th**	**5th**	**6th**

Th03-1	Yes ^1^	Yes ^1^	(*)	Yes ^1^	-
Th03-2	Yes ^1^	(*)	Yes ^1^	-	-
Th03-3	Yes ^1, 2^	Yes ^1^	Yes ^1, 2^	-	-
Th03-4	Yes ^1^	No	Yes ^1^	-	-

**Thies Clima 4.3350**					

**Anemometer**	**Maintenance before Calibration**				

	**2nd**	**3rd**	**4th**	**5th**	**6th**

Th50-1	No	No	No	-	-
Th50-2	No	Yes ^1^	(*)	-	-
Th50-3	(*)	(*)	(*)	-	-
Th50-4	No	No	No	-	-

**Anemometer**	**Maintenance before Calibration**				

	**2nd**	**3rd**	**4th**	**5th**	**6th**

LK-1	No	Yes ^1^	Yes ^1^	-	-
LK-2	No	Yes ^1^	No	-	-
LK-3	No	Yes ^1^	No	-	-
LK-4	No	Yes °	No	-	-
LK-5	No	No	No	-	-
LK-6	No	No	No	-	-
LK-7	No	Yes ^1^	Yes	-	-

(*) No information is available with regard to any possible maintenance before the calibration.

° No information is available with regard to the maintenance performed to the anemometer, but probably change of bearings;

1 Change of bearings;

2 Change of the cups' rotor.
